# Lameness and Hoof Disorders in Sheep and Goats from Small Ruminant Farms in Selangor, Malaysia

**DOI:** 10.3390/ani15131858

**Published:** 2025-06-24

**Authors:** Fatini Dayana Binti Rashid, Siti Nabilah Binti Mohd Roslan, Jacky Tan Lit Kai, Afida binti Ahmad Tajuddin, Siti Zubaidah Ramanoon, Azalea Hani Othman, Mohammed Babatunde Sadiq

**Affiliations:** 1 Department of Farm and Exotic Animal Medicine and Surgery, Faculty of Veterinary Medicine, Universiti Putra Malaysia, Serdang 43400, Selangor, Malaysia; fatinidayana@upm.edu.my (F.D.B.R.);; 2 Department of Veterinary Pathology and Microbiology, Faculty of Veterinary Medicine, Universiti Putra Malaysia, Serdang 43400, Selangor, Malaysia; azalea@upm.edu.my

**Keywords:** small ruminants, lameness, hoof disorders, animal welfare, risk factors

## Abstract

Hoof disorders in small ruminants pose significant challenges to animal welfare and farm productivity. This study presents the first attempt to determine the prevalence of lameness and hoof disorders and their associated risk factors in goat and sheep farms in Selangor, Malaysia. Locomotion scores were collected from 226 animals (126 sheep and 100 goats) across 10 farms. A hoof examination was conducted, and hoof lesions were identified through detailed photographic evaluation. On-farm assessments and interviews were conducted to gather information on management practices from the farms. Data were analysed using descriptive statistics, bivariate analysis, and logistic regression models. The prevalence of lameness was 42.8% (95% CI 34.2 to 51.9) in sheep and 23.0% (95% CI 16.3–38.4) in goats. Significant variation (*p* > 0.05) in lameness prevalence was observed across farms, ranging from 26.7% to 61.5% in sheep and 7.7% to 30.8% in goat farms. The majority of lameness and hoof lesions were observed in the hindlimbs of both species. The prevalence of hoof disorders was 91.3% (95% CI 84.6–95.4) in sheep and 43.0% in goats (95% CI 21.4–58.0). The predominant hoof disorders were overgrown wall horn, white line disease, sole bruise, and wall fissures. No hoof affections of infectious origin were observed in the sampled animals. Risk factors for lameness and hoof lesions in sheep included pregnancy, semi-intensive management, and breeds other than Damara. Higher odds of lameness were observed in exotic goat breeds and those with overgrown wall horns. In conclusion, this study revealed a high prevalence of lameness and hoof disorders in goat and sheep farms, highlighting the need to address these important welfare and economic issues. While the identified risk factors could be considered for the management of hoof disorders in small ruminant farms, a larger sample size that is representative of the sheep and goat population is recommended for more generalizable results.

## Simple Summary

Hoof disorders are major causes of lameness and among the most significant welfare and economic concerns in small ruminants. In this study, we present the first attempt to explore hoof disorders affecting sheep and goat populations in Selangor, Malaysia. We found that lameness and hoof disorders were more common in sheep relative to goats, with factors such as type of management, breeds and pregnancy status playing a significant role. Surprisingly, only non-infectious types of hoof disorders were detected in the affected animals. Our results highlight the need to develop effective lameness-preventive measures in the studied farms.

## 1. Introduction

Lameness encompasses gait and posture abnormalities or discomfort following the presence of foot or limb lesions [1]. Lameness and foot diseases are important causes of poor animal welfare in goats and sheep [2,3]. Studies conducted in Greece and New Zealand revealed that foot disorders affect the economic viability of small ruminant farms by reducing reproductive performance and increasing the risk of culling [2,4]. The economic consequences of hoof disorders in small ruminant farming are profound, escalating veterinary costs, reducing reproduction rates, and impairing meat or milk production [5].

As a multifactorial disorder, various aetiologies ranging from traumatic injuries and metabolic diseases may lead to foot-related lameness [2,4]. These conditions are broadly categorised as infectious and non-infectious hoof diseases [6,7]. Infectious hoof diseases, often caused by bacteria or fungi, have the potential to spread rapidly within goat and sheep populations. Meanwhile, non-infectious hoof diseases result from both animal-level (i.e., parity, body condition, age) and farm-level factors such as inadequate diets, suboptimal hoof care, and environmental conditions [8,9,10].

The causes of goat lameness are similar to those recorded in sheep [3], with hoof disorders such as white line disease, interdigital dermatitis, foot rot, and heel horn erosion reported in both species [6,7]. Nevertheless, the extent of these problems may differ between goats and sheep. For instance, the prevalence of hoof lesions in sheep and goats from Brazilian farms was 19.4% (170/876) and 17.9% (52/289), respectively, with a herd prevalence between 5.8% and 33.8% [11]. In the UK, the average lameness level was relatively lower, ranging from 4.9% to 10.0% [12], which is lesser compared to dairy goats at 19.2%, with a high range of 0 to 52% between farms [13]. Large differences were also observed in goat lameness prevalence (6.7–25.5%) in New Zealand, with hoof lesions found in 65.5% of trimmed goats [3,14]. This variation in lameness levels is linked to the preponderance of risk factors in the studied locations.

Despite the global significance of these challenges, comprehensive data on lameness and hoof diseases in small ruminants, especially in specific regions like Malaysia, remain notably limited. Farm animal lameness studies in Malaysia have focused mainly on bovine species, especially dairy cattle [15,16], with a prevalence ranging from 19.0 to 33.0%. However, there is limited to no available data on lameness in sheep and goat farms in Malaysia except for a few case reports [17,18].

Welfare assessment studies on ruminant farms revealed that farmers usually underestimate lameness prevalence in their farms and only address the problem when the condition is severe [19,20,21]. This knowledge gap hinders the development of targeted strategies for effective management, impeding efforts to maintain optimal welfare, support sustainable farming practices, and minimise economic losses in the Malaysian small ruminant industry. Factors such as climate, environmental conditions, and local farming practices in regions like Malaysia may influence the occurrence and severity of these issues in small ruminants [5]. Research endeavours addressing the hoof health of small ruminants in Malaysia are imperative for the development of targeted strategies to mitigate these challenges. Therefore, this study investigates the prevalence of lameness and hoof diseases and their potential risk factors in sheep and goat farms in Selangor, Malaysia.

## 2. Materials and Methods

### 2.1. Ethical Approval

The protocol for this study was reviewed and approved by the Institutional Committee for Animal Care and Use (UPM/IACUC/AUP-U023/2023), Universiti Putra Malaysia.

### 2.2. Farms and Animals

This cross-sectional study was conducted on 10 farms, specifically involved in meat production, in five districts of Selangor, Malaysia. The farms included foster farms of the Universiti Putra Malaysia’s Veterinary Hospital (UVH) and a few other commercial farms in Selangor. The farms were recruited through personal requests and were considered suitable for the study if they had either a loose or indoor housing system and a herd size of more than 20 goats or sheep. The herd size on the sheep and goat farms ranged from 25 to 200 animals.

The sample size was calculated for each species under investigation using the formula by Thrusfield [22]. The parameters considered include an expected prevalence of 9% in goats [23] and 12% in sheep [12], an animal population of 5000 for both species, a confidence interval of 95%, and an error rate of 5%. The calculated sample sizes were 126 and 111 for sheep and goats, respectively.

Prior to the farm visits, the researcher communicated with the farmer to gather information on the herd size, purpose of production, breed, and number of adult animals. Computer-generated numbers were assigned to the animals on each farm. Subsequently, 20% of animals in each farm were then selected randomly for the study using Quirk’s method with little modification, which entailed accounting for specific factors that may introduce sampling bias [24]. In this study, given the variety of breeds in the sheep and goat farms, breed was identified as the animal-based factor with the strongest variation across farms. In farms with more than one breed of animals, the factor was considered during sample selection, ensuring that the breeds were balanced as much as possible. Upon visiting the farm, the preselected animals were inspected by a veterinarian for any apparent disease aside from lameness and hoof diseases, such as diarrhoea, mastitis, pregnancy toxaemia, and general poor health status. Animals with any health problems were replaced accordingly. The farms were visited from 5 August 2023 to 21 September 2023.

### 2.3. Farm Characteristics

Data for farms’ characteristics, herd management, and housing were gathered using a structured questionnaire administered via a face-to-face interview with farmers. The instrument consisted of two sections. Section 1 focused on the farmers’ information such as educational qualification, age, experience in livestock farming, and knowledge of lameness and hoof health in farmed animals. Meanwhile, Section 2 covered farm management and potential risk factors for lameness and hoof diseases, such as the management system, housing design, feeding system and regimen, stage of production-based feeding regime, hoof trimming practices, frequency of cleaning the pen, and presence of footbaths.

#### 2.3.1. Locomotion Scoring

Locomotion scoring (LS) was conducted by visual observation of the animal’s gait based on a five-point LS system developed by Deeming et al. [25]. The procedure, designed to ensure accuracy and reliability, entailed allowing the animals (goats and sheep) to complete at least 3 or more strides at a normal pace on a flat surface. Animals exhibiting an LS of ≥2 were categorised as lame, thereby establishing a standardised criterion for lameness determination. Before main data collection, 2 assessors were trained on how to perform the locomotion scoring by observing 15 goats in the institutional veterinary hospital. Both inter-rater and intra-rater reliability were computed, and the results were acceptable with a Kappa coefficient exceeding 0.70 for all assessments.

#### 2.3.2. Animal Characteristics

Animal-based measures were systematically examined as part of the welfare assessment in the selected farms. Body condition score (BCS) was appraised with a refined approach based on the method developed by Ngwa et al. [26]. The scoring system incorporated 0.5 increments, with BCS ≤ 2.0 categorised as poor condition, BCS ≥ 2.5 and ≤4 as good condition, and BCS ≥ 4 as over-conditioning. Age was determined using dentition, based on the degree of wear and development of animals’ teeth [27]. Other animal characteristics recorded were breed, pregnancy status, and hock condition.

#### 2.3.3. Hoof Examination

Hoof conditions were recorded in detail by measuring fore and hind claws, the degree of overgrown wall horn, and the presence of slipper foot. We measured the medial and lateral claws of all hooves using a measuring tape as described by Bhardwaj et al. [28].

The claw characteristics measured include toe length, heel height, sole length, and sole width. Toe length entails the distance between the digit’s apex and the dorsal coronary band. Heel height was defined as the vertical distance between the sole and the coronary band. Sole length entails the length between the bulb (i.e., aspects in contact with the floor) and the abaxial aspect of the claw. Meanwhile, sole width constitutes the largest distance between the axial and abaxial walls at the sole–heel junction.

Using a Canon Power Shot (Zoom lens 5 × 15, Canon Malaysia, Selangor, Malaysia), two pictures of each foot were taken encompassing the later and dorsal aspects, to determine if wall horn overgrowth and slipper foot were present. Overgrowth was identified based on the degree of sole surface covered by overgrown wall horn, which was classified using a 3-point scale. A foot was identified as a slipper foot if either one or both claws had chronic overgrowth—defined as a long curling toe [29].

#### 2.3.4. Claw Lesions

Claw lesions were identified using a modified claw card by Lottner [30], which entailed focusing mainly on the claw zones and hoof capsule, without examining regions above the coronet. The same veterinarian performed the claw lesion examination on all farms. All sampled animals were inspected for claw lesions irrespective of their locomotion scores. For each animal, all four feet were observed and classified into medial and lateral claws. For each claw, the listed lesions were ticked if present. Briefly, the list of claw lesions comprised horn/wall fissure, horn separation, granulomatous lesion, heel horn erosion, chronic laminitis, interdigital phlegmon, toe/sole abscess, toe/sole ulcer, and sole haemorrhage. In addition, conditions affecting the interdigital space include mild-to-severe dermatitis. All results were verified using the pictures taken during claw examination, and a senior veterinarian was called upon to clarify doubtful findings.

### 2.4. Data Analysis

The data collected were managed in Microsoft Excel 2019. The prevalence of lameness and specific claw lesions, as well as 95% confidence intervals (CIs), were computed using descriptive statistics, the exact binomial method, and an Excel calculator. Potential risk factors for both outcomes were evaluated using Pearson’s chi-squared test and odds ratio (OR) when necessary, in IBM SPSS Statistics Version 29 (IBM Corp., Armonk, NY, USA). A factor was considered statistically significant when the *p*-value was <0.05.

Two outcome measures, lameness and the presence of hoof disorders on sheep and goat farms, were tested using logistic regression models. Given the nature of the data and assessment of animal- and farm-level explanatory variables, multi-level mixed-effects logistic regression models were built to identify the predictors of lameness and hoof disorders [31]. The explanatory variables (independent factors) at the farm level were footbath practices, hoof trimming, management systems, stage of production-based feeding, frequency of cleaning, and years of experience. Meanwhile, the animal-level factors included breeds, pregnancy status, gender, age, BCS, and the presence of overgrown hooves. All explanatory variables were introduced as main effects, while farm was considered a random effect. A backward elimination method was applied to reduce the multivariable models, and variables presenting a *p*-value < 0.05 were retained. A confounding factor was identified when its removal from the model led to at least a 30% alteration in the estimate of other significant predictors. Model fit was determined based on the lowest value of Akaike’s information criterion upon comparing all the available multivariable models.

## 3. Results

### 3.1. Descriptive Findings

Overall, ten farms were included in this study, corresponding to four sheep farms and six goat farms in Selangor, Malaysia (Table 1). As for the sheep farms, the number of animals in each farm ranged from 110 to 200. Two farms each practised intensive and semi-intensive systems. All the sheep farms had a raised housing and flooring system, but only one farm had access to a pasture area and an external exercise yard. The frequency of cleaning ranged from daily to thrice per day. Hoof trimming was practised when deemed necessary following visual assessment on Farm A, once every 3 months on Farm B, once or twice a year on Farm C, and not practised on Farm D. Farmers’ years of experience in sheep farming ranged from 6 to 12 years.

Likewise, the herd size differed across goat farms, with three each having >50 and <50 animals. Three farms each practised intensive system and semi-intensive management systems. The housing design in most farms was raised housing constructed with wooden materials, except for Farm 3 with a concrete floor. Two farms had access to a pasture area and an external exercise yard. The frequency of cleaning ranged from twice to thrice per day. The feeding regimen comprised mainly of forage and pellets provided on two farms, while Napier, soy, palm leaves, palm kernel cake, and silage were given on other farms in different mixes. None of the farms practised hoof trimming or had a vaccination programme. However, two farms had a deworming programme. Farmers’ years of experience in goat farming ranged from 10 to 20 years.

### 3.2. Animal-Level Characteristics

A total of 126 sheep and 100 goats were sampled from the studied farms. Table 2 summarises the animal characteristics. The sheep’s age ranged from 1 to 4 years old, comprising various breeds such as Black Belly, Santa Ines, Merino, Damara, Dorper, and Morada Nova. Most of the sampled sheep were female (117/126; 92.8%), with a mean BCS of 3.0, and 14/117 of the ewes were pregnant. In addition, the majority had a normal/healthy hock condition (72/117) relative to those with mild hair loss (50/126) and severe injury (ulcer or open wound) (4/126).

For the sampled goats, the mean age (SD) and BCS were 2.35 (±0.58) years old and 2.73 (±0.50), respectively. Sampled goats were generally more even in terms of gender, comprising 46.0% and 54.0% male and female animals, respectively. A slightly higher proportion was local breeds (i.e., Katjang) (51.0%) compared to exotic breeds (49.0%). Only 20 goats were pregnant. Hock examination revealed 85.0% with normal/healthy hock (85/100), 10.0% with mild hair loss (10/100), and 5.0% with an ulcerated wound on the hock area (5/100).

### 3.3. Prevalence of Lameness

The prevalence of lameness in sheep was 42.8% (54/126, 95% CI 34.2 to 51.9%). Most of the lame animals were mildly lame with a locomotion score (LS) of 1 (33/126, 26.1%), followed by moderately lame (i.e., LS2, 19/126; 15.0%) and severely lame (LS3, 3/126; 2.4%). As shown in Table 3, a significant variation in lameness prevalence was observed across farms, with Farm 4 recording the highest prevalence at 61.5% (95% CI 40.6 to 79.1), 56.3% (95% CI 37.9 to 73.2) on Farm 2, 31.6% (95% CI 18.0 to 48.8%) on Farm 1, and 26.7% (95% CI 12.9 to 46.2%) on Farm 3.

Among the sampled goats, the prevalence of lameness was 23.0% (23/100; 95% CI 16.32–38.41), with the majority being moderately lame (LS 3, 19/100, 19.0%), followed by mild lame (LS2; 2/100, 2.0%) and severely lame goats (LS2; 2/100, 2.0%). Farm 3 had the highest lameness prevalence at 30.8% (8/25), followed by Farm 5 at 26.9% (7/26), Farm 2 at 25.0% (3/12), Farm 1 at 20.8% (4/24), and Farm 4 at 7.7% (1/12), indicating significant variation across farms.

As shown in Table 4, a significantly higher proportion (*p* < 0.05) of lame sheep were affected on the left hindlimb (52.0%), followed by the right hindlimb (38.0%), and least on the forelimbs (12.0%). Likewise, a significant proportion (*p* < 0.05) of affected goats had left hindlimb lameness (47.8%; 11/23) and right hindlimb lameness (30.4%; 7/23) compared to those affecting the left forelimb (2/23; 8.7%) and right forelimb (3/23; 13.0%).

### 3.4. Prevalence of Claw Lesions

Table 5 depicts the prevalence of claw lesions in sheep at animal and farm levels. The overall prevalence of claw lesions was 91.3% (95% CI 84.6–95.4), indicative of poor hoof health in the studied farms. Overgrown claw was detected in 90.5% (95% CI 83.6–94.8) of the animals, whereas the prevalence of white line disease and sole bruise was 39.6% (95% CI 31.2–48.8%) and 10.3% (95% CI 5.8–17.3%), respectively. Hoof affections of infectious origin were not observed in the sampled sheep on the four farms. Farm 1 had the highest prevalence at 100.0% relative to Farm 2 with the lowest prevalence at 72.0%.

The overall prevalence of hoof lesions in goats was 43.0% (43/100; 95% CI 21.41–58.05%), with a significant variation across farms (Table 6). Overgrown claw/slipper foot was observed in 28.0% (95% CI) of the animals, 12% had WLD/shelly hoof, 6% had sole bruises, and 5% had wall fissures. None of the sampled goats had claw lesions of infectious origin, suggesting a low prevalence of such hoof affections in the sampled farms.

**Figure 1:**
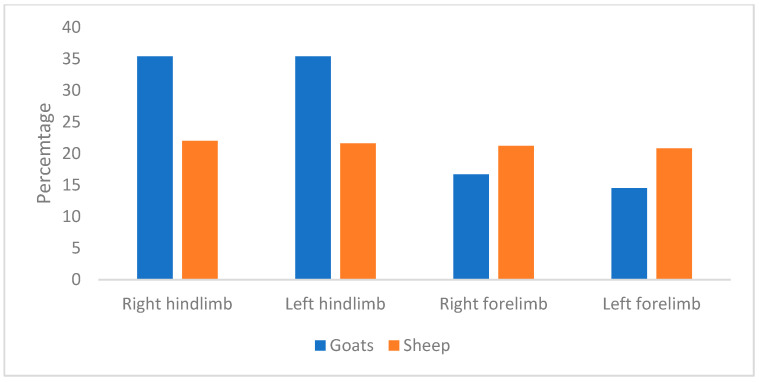
Percentage of sampled goats and sheep affected with hoof disorders.

**Figure 2:**
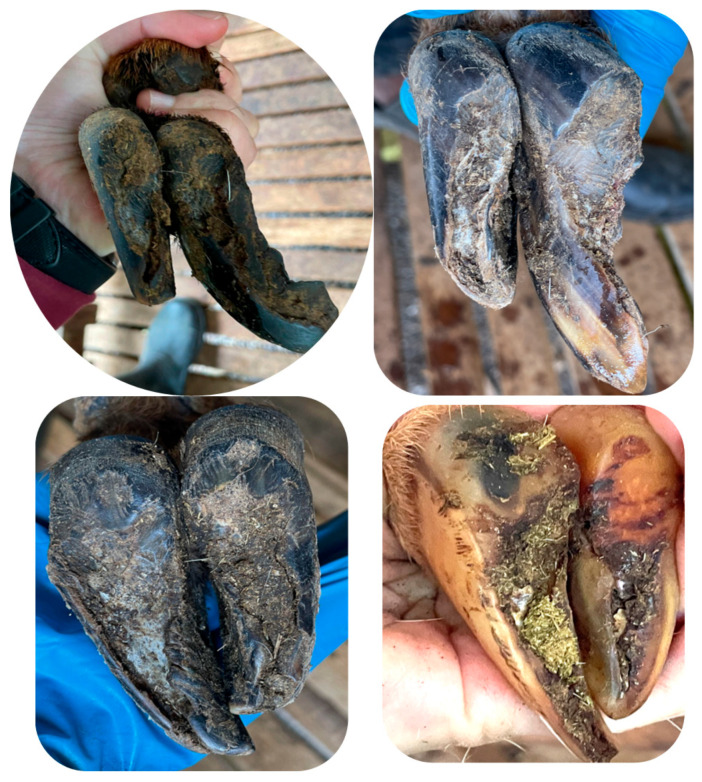
Images of some of the predominant claw lesions identified in the study population. First row, left side: overgrown foot; first row, right side: wall fissure; second row, left side: white line disease/slipper foot; second row, right side: sole bruise.

In terms of the affected foot in sheep, similar proportions (*p* > 0.05) of hoof lesions were observed on the right hindlimb, left hindlimb, right forelimb, and left forelimb at 22.0%, 21.6%, 21.2%, and 20.8%, respectively (Figure 1). In contrast, significantly higher proportions of claw lesions in goats were observed on the right and left hindlimbs (35.4% vs. 35.1%) compared to the right (16.7%) and left forelimbs (14.5%). Figure 2 depicts some of the hoof disorders.

### 3.5. Factors Associated with Lameness and Claw Lesions in Sheep and Goat Farms

In the univariable model for association analyses between lameness and explanatory variables, the significant factors include hoof trimming (*p* = 0.034), pregnancy status (*p* = 0.009), years of experience (*p* = 0.034), availability of footbath (*p* = 0.044), and management system (*p* = 0.001). In the multivariable model, only pregnancy status (*p* = 0.026) and management system (0.027) were significant. Specifically, increased odds of lameness were observed in pregnant ewes (OR = 5.02; 95% CI 1.21–20.72) and semi-intensively managed farms (OR = 3.09; 95% CI 1.14–8.38) (Table 7).

As for the prevalence of hoof lesions on sheep farms, the univariable model depicted four significant factors, namely management system (*p* = 0.013), stage of production-based feeding (*p* = 0.001), and breeds (*p* = 0.005). In the multivariable model, only the breed of sheep demonstrated a significant association (*p* = 0.04) with the prevalence of hoof lesions. Breeds other than Damara (OR = 12.92, 95% CI 1.06–160.29 were more likely to develop at least one hoof lesion relative to the Damara breed (Table 8).

In the multivariate model, the prevalence of lameness on goat farms was influenced by the presence of claw lesions and breeds (Table 9). Goats with overgrown claws (OR = 8.43; 95% CI 2.31–30.75) and exotic breeds (OR = 2.45; 95% CI 1.08–6.21) demonstrated a higher risk of lameness relative to those without claw lesions and local breeds, respectively. None of the tested factors was associated with the prevalence of hoof lesions.

## 4. Discussion

In this research, we present the first detailed information on the hoof health status of small ruminant farms in Selangor, Malaysia. The prevalence of lameness in sheep is relatively higher (42.8%) compared to the estimate in goats (23.0%), with significant variation across farms. As for sheep, the animal-level prevalence is higher compared to the reports on sheep flocks in Greece at 9.0% [23], Nigeria at 7.2% [32], the United Kingdom at 10.0% [12], and Brazil at 19.4% [11]. In contrast, the prevalence of lameness on our goat farms is similar to reports in other countries such as Brazil at 17.9% [11], UK at 19.2% [13], and New Zealand at 25.5% [14]. Factors such as the definition of lameness, diagnostic methods, and management systems may have contributed to these findings.

Given the present study design, the underlying reasons for the higher prevalence of lameness in sheep relative to goats cannot be ascertained. Nevertheless, the higher proportion of sheep affected with hoof lesions compared to goats (91.3% vs. 43.0%) may have contributed to this finding. Our result is higher compared to a recent study in New Zealand at 65.5%; however, only trimmed goats were observed in their study [3].

Similar findings were observed in the sampled goat and sheep population in terms of specific hoof affections and limbs affected. Only claw lesions of non-infectious origin were observed in both species, especially overgrown claw/slipper claw, WLD, and sole bruise. These findings are consistent with reports in Swiss and UK dairy goats, whereby approximately 100% of examined animals had wall horn overgrowth [13].

As evidenced in the present study, the presence of overgrown claws was the only factor that increased the risk of lameness in sampled goats. Our previous research on bovine lameness in Selangor and Peninsular Malaysia showed that cows with poor hoof conformation are more vulnerable to hoof lesions and lameness [15,16]. Overgrown wall horns can trigger claw deformation and painful claw lesions, leading to abnormal gait and stress on the supportive apparatus, tendons, ligaments, and joints [6]. Furthermore, a thermographic assessment of goats’ claws revealed a strong correlation between deformation, wall horn overgrowth, and deep inflammation [33]. These events culminate in lameness, particularly in the long term, highlighting the need to address the problems. It was noted that most of the claw lesions and lameness were recorded on the hindlimbs (78.2%) compared to the forelimbs (21.7%). In dairy cows, hindlimb lameness is more common due to the disproportionate weight distribution, with most weight directed unto the rear aspect of the animal [34]. Although a similar mechanism may explain the occurrence in dairy goats and sheep, evidence is lacking to support this hypothesised event.

Hoof lesions of infectious origin were not observed in any of the goat and sheep farms. Given that a case report in Selangor described the occurrence of foot rot in Boer goats [17] and seropositivity for caprine arthritis encephalitis lentivirus [18], our finding reflects the need for further research and larger studies covering most farms in Selangor and other Malaysian states. Nevertheless, the result suggests a low prevalence of infectious hoof lesions in the studied farms, which is similar to the reports from all bovine lameness investigations in Malaysia [15,16]. Climates such as dry and hot conditions, which were predominant during the sampling period, may equally play a role in this finding, as such conditions decrease the longevity of *Dichelobacter nodosus* (*D. nodosus*—the aetiologic agent for foot rot) outside the host’s environment, thereby lowering the transmission rates among sheep [35].

On the other hand, we found a high prevalence of overgrown wall horns in goat and sheep farms, possibly linked to confinement in pens with hard surfaces (slated wood), which reduces horn wear. Hard flooring surfaces amplify the physical impact of severe load bearing on feet and tend to irritate the corium and promote hoof growth, which causes overgrowth and overloading of the affected claws. This was particularly evidenced in one of the goat farms (Farm 3) with concrete flooring, recording the highest lameness prevalence among the sampled farms. Overall, lesions affecting the wall horn of small ruminants have been linked to diverse factors such as the quality of ground and bedding, nutritional deficiencies, and genetic factors [2,36]. Hill et al. [29], on the other hand, posited that external factors are the underlying events leading to wall horn separation rather than metabolic events.

In our study, specific animal- and farm-level factors were observed to heighten the risk of lameness and hoof lesions, especially in sheep. For instance, pregnant ewe demonstrated a significantly higher risk of lameness. While the association between pregnancy and lameness is underreported in small ruminants, Chapinal et al. [37] found that gait scores and weight distribution of the legs were affected when cows were in their late trimesters. Sheep other than Damara and exotic goats demonstrated a higher risk of lameness and claw lesions, respectively. Prior research has shown that sheep with lighter-coloured hooves were more likely to suffer from sole bruises and white line disease compared to those with darker claws [38]. Delayed production in hardness (promoted by intracellular disulphide-bonding) may be associated with the presence of a reducing chemical environment produced by the antioxidant properties of melanin [39], which aids in the natural wear and tear in small ruminants with dark-coloured hooves. This might contribute to our finding as both Damara and local breeds in our study had darker claws relative to other breeds of sheep and goats.

At the farm level, a higher prevalence of lameness and claw lesions were observed in sheep herds managed semi-intensively compared to those reared intensively. This finding is surprising given the lower risk of overgrown hooves in extensive grazing systems, aligning with natural hoof-wearing due to prolonged contact between the hoof and ground surfaces [40]. Likewise, animals that graze on alpine pastures had the lowest amount of wall horn overgrowth due to prolonged contact between the hoof and hard soil while those confined indoors had the most [36]. An indoor area with wooden floors, as observed in the present study farms, does not promote sufficient hoof wear given the lower friction at the claw–floor interface [36]. The limited walking distance and restricted movement in indoor housing are also expected to establish conditions for overgrown hooves, which are more susceptible to lameness. Nevertheless, one reason for the higher risk of lameness in semi-intensive systems relative to intensive management might be increased exposure to an external environment with a high risk of traumatic injuries and foreign bodies penetrating the sole, leading to foot-related lameness [3].

Hoof trimming was not statistically proven to be protective against lameness and hoof disorders in the studied farms. In UK sheep farms that were managed extensively and highly affected by infectious hoof lesions, foot trimming was found to be associated with a higher prevalence of lameness [10,41,42]. Comparatively, our studied farms were more intensive and the prevalent hoof disorders were non-infectious types, suggesting the need for routine hoof trimming. This was demonstrated in our previous studies on bovine lameness, whereby hoof trimming was identified as pertinent either as a preventive programme or treatment modality for non-infectious hoof lesions such as sole haemorrhage, sole ulcer, and white line disease [43,44,45]. These hoof disorders are similar to those observed in the present study, particularly sole bruises, sole haemorrhage, and white line separation. Nevertheless, larger cross-sectional studies are required to ascertain the benefits of these practices in the management and control of ovine lameness in Malaysian farms.

There was no association between footbath practices and the prevalence of lameness and hoof disorders in this study, as depicted in the final regression model. Prior research has shown mixed effects of foot bathing on the management of contagious ovine digital dermatitis, foot rot, and non-infectious hoof lesions such as WLD and sole bruises [2,46]. Studies in the UK found that routine foot bathing exacerbated the occurrence of granulomas and shelly hoof in sheep [41], and the practice was unable to reduce the incidence of non-infectious hoof lesions [12,42,47]. The sheep farms in our study were not practising routine foot bathing, but rather did so only after hoof trimming and when animals experienced gait disturbance. Since infectious hoof disorders were not detected in any of the studied farms and given the present study design, the rationale for practising foot bathing in some farms and the impact on hoof health is not well understood. Further studies are required to bridge this critical research gap.

## 5. Limitations

The limitations of this study include the relatively small sample size and few farms, which may not represent the population of small ruminants in Selangor. Nevertheless, our intention was to ascertain if lameness is a common problem in the studied farms rather than generalising the findings. Given the cross-sectional design used in this study, definite causes of lameness and hoof lesions could not be determined. Our results are limited to the current hoof health status in goat and sheep farms, as well as the associated factors. As a result, causal relationships cannot be drawn from this study.

## 6. Conclusions

In conclusion, the prevalence of lameness and hoof lesions was relatively higher in sheep farms (42.8% and 91.27%) compared to goat farms (23.0% and 43.0%). Non-infectious hoof affections such as WLD, sole bruises, and overgrown claws were mainly responsible for lameness in both ruminant species. Semi-intensive management, breed, and pregnancy status are significant factors for lameness and hoof disorders in sheep and goat farms. Studies with a larger sample size are required to ascertain the benefits of hoof trimming and foot bathing practices as lameness management strategies in Malaysian small ruminant farms. The present results reflect the urgent need to address lameness in the studied farms, highlighting potential strategies for the control and prevention of these critical health and welfare issues.

## Figures and Tables

**Table 1:** Farm characteristics of the small ruminant farms included in this study.

	Management System	Herd Size	Feeding	Housing and Flooring	Pasture Access	Exercise Area	Deworming Routine	Vaccination Routine	Hoof Trimming	Footbath
				Goat farms						
**Farm 1**	Intensive	>50	Forage, pellet	Wooden Raised	No	No	No	None	None	None
**Farm 2**	Semi-intensive	<50	Soy, pellet, palm leaves	Wooden Raised	Yes	Yes	No	None	None	None
**Farm 3**	Intensive	>50	Napier, pellet	Wooden and concrete floor	No	No	Yes	None	None	None
**Farm 4**	Intensive	<50	Forage, pellet	Wooden Raised	No	No	No	None	None	None
**Farm 5**	Semi-intensive	>50	Forage, Pellet, Silage, PKC	Wooden Raised	Yes	Yes	Yes	None	None	None
**Farm 6**	Semi-intensive	>50	Forage, Pellet, Silage, PKC	Wooden Raised	Yes	Yes	Yes	None	None	None
	Sheep farms									
**Farm 1**	Intensive	150	Forage, Pellet, Silage	Wooden Raised	No	No	Yes	Yes	Twice/year	No
**Farm 2**	Semi-intensive	200	Soy, pellet, palm leaves	Wooden Raised	Yes	No	Yes	Yes	Twice/year	Yes
**Farm 3**	Intensive	110	Forage, Pellet, Silage, PKC	Wooden raised	No	No	Yes	Yes	Once/three months	Yes
**Farm 4**	Semi-intensive	200	Forage, Pellet, Silage, PKC	Wooden raised	Yes	No	Yes	Yes	No	No

**Table 2:** Animal-level characteristics.

Characteristics	Sheep			Goat	
	**Frequency**	**%**		**Frequency**	**%**
**Age**			**Age**		
≤2 years	41	32.5	≤2 years	62	62.0
>2 years	85	67.5	>2 years	38	38.0
**Mean age (SD)**			**Mean age (SD)**	2.35 (0.58)	
**Breed**			**Breed**		
**Damara**	49	38.8	**Katjang**	51	51.0
**Others**	77	61.2	**Exotic**	49	49.0
**Gender**			**Gender**		
**Male**	9	7.1	**Male**	46	46.0
**Female**	117	92.9	**Female**	54	54.0
**Hock condition**			**Hock condition**		
**Normal**	72	57.1	**Normal**	85	85.0
**Mild hair loss**	50	39.6	**Mild hair loss**	10	10.0
**Severy injury**	4	3.3	**Severy injury**	5	5.0
**BCS**			**BCS**		
<3	64	50.7	<3	45	45.0
≥3	62	49.2	≥3	55	55.0
**Mean BCS (SD)**	3.0		**Mean BCS (SD)**	2.75 (0.50)	
Pregnancy status *			Pregnancy status *		
**Pregnant**	14	11.1	**Pregnant**	20	20.0
**Not Pregnant**	103	88.9	**Not Pregnant**	34	34.0

* Pregnancy status only computed for female animals. Others refer to Black Belly, Santa Ines, Merino, Dorper, and Morada Nova. Exotic breeds for goats include Boer, Jamnapari and Saanen. BCS = body condition score, SD = standard deviation.

**Table 3:** Overall prevalence of lameness in sheep and goats at animal and farm levels.

	Number of Lame	Number of Non-Lame	Total	Prevalence (%)	95% Confidence Interval (%)
**Sheep**					
**Farm 1**	12	26	38	31.58	18.03–48.79
**Farm 2**	18	14	32	56.25	37.88–73.17
**Farm 3**	8	22	30	26.67	12.98–46.18
**Farm 4**	16	10	26	61.54	40.57–79.09
**Overall**	54	72	126	42.86	34.19–51.98
**Goat**					
**Farm 1**	4	20	24	20.8	14.62–34.05
**Farm 2**	3	9	12	25.0	15.21–36.45
**Farm 3**	8	17	25	30.8	20.41–42.44
**Farm 4**	1	12	13	7.7	2.56–14.24
**Farm 5**	7	19	26	26.9	12.31–38.02
**Overall**	23	77	100	23.0	16.32–38.41

**Table 4:** Distribution of lame foot in 126 sheep and 100 goats.

	Sheep		Goat	
**Affected limb**	**Lame**	**%**	**Lame**	**%**
**Left Forelimb**	2	4.0	2	8.7
**Left Hindlimb**	28	52.0	11	47.8
**Right Forelimb**	4	8.0	3	13.0
**Right Hindlimb**	20	38.0	7	30.4
**Total**	54	126	24	100

**Table 5:** Distribution of hoof disorders in 126 sheep from four farms.

Types of Hoof Conditions					Total	Prevalence (95% CI)
	Farm 1	Farm 2	Farm 3	Farm 4		
**Overgrowth**	38	22	29	25	114	90.48 (83.62, 94.77)
**WLD/Shelly hoof**	16	6	10	18	50	39.68 (31.19, 48.80)
**Sole bruise**	13	0	0	0	13	10.32 (5.83, 17.33)
**Normal hooves**	0	9	1	1	11	8.73 (4.65, 15.44)
**No. of animals with hoof conditions**	38	23	29	25	115	91.27 (84.56, 95.35)
**Total no. of animals**	38	32	30	26	126	
**Prevalence of by farm (95% CI)**	100.0 (88.5, 100)	71.9 (53.3, 85.6)	96.7 (80.95, 99.8)	96.2 (78.4, 99.8)	91.3 (84.6, 95.4)	

Note: percentage exceeds 100% due to more than 1 hoof condition per sheep.

**Table 6:** Distribution of hoof disorders in six goat farms.

Types of Hoof Conditions	Number of Animals					Total	Prevalence (95% CI)
	Farm 1 (*n* = 20)	Farm 2 (*n* = 20)	Farm 3 (*n* = 20)	Farm 4 (*n* = 20)	Farm 5 (*n* = 20)		
**Overgrowth**	9	3	7	3	6	28	28.02 (12.01–38.2)
**WLD/Shelly hoof**	4	1	3	2	2	12	12.01 (5.10–18.42)
**Sole bruise**	2	1	2	0	1	6	6.12 (2.82–14.04)
**Wall fissure/crack**	2	0	2	0	1	5	5.32 (1.84–11.08)
**No. of animals with hoof conditions**	13	3	14	4	9	43	43 (21.41–58.05)
**Prevalence of by farm (95% CI)**	62.8 (44.71–86.20)	16.7 (9.48–34.01)	69.2 (41.54–83.22)	19.2 (8.42–30.12)	46.2 (24.82–68.22)		

Note: percentage exceeds 100% due to more than 1 hoof condition per goat.

**Table 7:** Multivariable model for factors associated with lameness in sheep (*n* = 126) from four farms.

Variables	Normal	Present	Prevalence (%)	95% CI	*p*-Value	Odds Ratio (95% CI)
**Pregnancy status**						
**Pregnant**	11	3	78.5	48.8–94.3	0.026	5.02 (1.21–20.72)
**Not pregnant**	39	64	37.8	28.6–47.10		Ref
**Management system**						
**Semi-intensive**	34	24	58.6	44.9–71.1	<0.027	3.09 (1.14–8.38)
**Intensive**	20	48	29.4	27.2–51.1		Ref
**Hoof trimming**						
**Yes**	38	62	38.0	28.6–48.2	0.71	1.23 (0.39–3.84)
**No**	16	10	61.5	40.7–79.0		Ref
**Presence of Footbath**						
**Yes**	8	22	26.6	12.9–46.1	0.77	0.85 (0.28–2.50)
**No**	46	50	47.9	37.7–58.3		Ref

Note: years of experience was excluded from the final model given its redundancy with the variable; hoof trimming.

**Table 8:** Multivariable model for factors associated with hoof lesions in sheep (*n* = 126) from four farms.

Variables	Normal	Present	Prevalence (%)	*p*-Value	OR (95% CI)
**Breed**					
**Damara**	10	39	79.5	0.04	Ref
**Others**	1	76	98.7		12.92 (1.06–160.29)
**Management system**					
**Semi-intensive**	8	24	75.0	0.21	0.13 (0.006–3.02)
**Intensive**	26	68	70.8		Ref
**Stage of production-based feeding**					
**Yes**	2	94	97.9	0.573	0.46 (0.033–6.56)
**No**	9	21	70.0		Ref

Ref = reference group, OR = odds ratio, CI = confidence interval.

**Table 9:** Multivariable model for factors associated with lameness in goats.

Variables	B	SE	Wald	*p*-Value	Odds Ratio (95% CI)
**Breed**					
**Katjang**	1.93	0.77	8.43	0.03	2.45 (I.14–6.30)
**Exotic**					Ref
**Overgrown hooves**					
**Present**	2.13	0.66	10.42	0.001	8.43 (2.31–30.76)
**Absent**					

B = regression coefficient, Ref = reference group, SE = standard error, CI = confidence interval.

## Data Availability

The data presented in this study are available on request from the corresponding authors due to confidentiality reasons.
